# Exploring the impact of the Baby-Friendly Hospital Initiative on trends in exclusive breastfeeding

**DOI:** 10.1186/1746-4358-4-11

**Published:** 2009-10-29

**Authors:** Sheryl W Abrahams, Miriam H Labbok

**Affiliations:** 1 Carolina Global Breastfeeding Institute (CBI), Department of Maternal and Child Health, Gillings School of Global Public Health, Rosenau Hall, The University of North Carolina at Chapel Hill, Chapel Hill, NC 27599-7445, USA

## Abstract

**Background:**

The Baby-Friendly Hospital Initiative (BFHI) seeks to support breastfeeding initiation in maternity services. This study uses country-level data to examine the relationship between BFHI programming and trends in exclusive breastfeeding (EBF) in 14 developing countries.

**Methods:**

Demographic and Health Surveys and UNICEF BFHI Reports provided EBF and BFHI data. Because country programs were initiated in different years, data points were realigned to the year that the first Baby-Friendly hospital was certified in that country. Pre-and post-implementation time periods were analyzed using fixed effects models to account for grouping of data by country, and compared to assess differences in trends.

**Results:**

Statistically significant upward trends in EBF under two months and under six months, as assessed by whether fitted trends had slopes significantly different from 0, were observed only during the period following BFHI implementation, and not before. BFHI implementation was associated with average annual increases of 1.54 percentage points in the rate of EBF of infants under two months (p < 0.001) and 1.11-percentage points in the rate of EBF of infants under six months (p < 0.001); however, these rates were not statistically different from pre-BFHI trends.

**Conclusion:**

BFHI implementation was associated with a statistically significant annual increase in rates of EBF in the countries under study; however, small sample sizes may have contributed to the fact that results do not demonstrate a significant difference from pre-BFHI trends. Further research is needed to consider trends according to the percentages of Baby-Friendly facilities, percent of all births occurring in these facilities, and continued compliance with the program.

## Background

Breastfeeding, especially exclusive breastfeeding (EBF), is one of the most effective preventive health measures available to reduce child morbidity and mortality [1]. The international Baby-Friendly Hospital Initiative (BFHI) was launched in 1991 by UNICEF and WHO to promote and protect maternal and child health by ensuring support for breastfeeding in maternity care facilities [2]. Since that time, more than 20,000 health care facilities in more than 150 countries around the world have achieved Baby-Friendly certification from their national certifying body (Labbok M, personal communication from global query carried out in 2006, [3]) by implementing the Ten Steps to Successful Breastfeeding and ending the practice of distributing free or low-cost breast milk substitutes [4,5].

Evidence from developed and developing countries indicates that the BFHI has had a direct impact on breastfeeding rates at the hospital level [6-11]. In a randomized controlled trial in Belarus, Kramer et al. noted improved rates of any and exclusive breastfeeding at 3 and 6 months and any breastfeeding at 12 months, in infants of mothers giving birth at hospitals randomized to follow BFHI policies, compared to those delivering at control hospitals [7]. A 2003 analysis of data from Swiss mothers demonstrated that rates of EBF for infants 0 to 5 months was significantly higher among those delivered in Baby-Friendly hospitals than in the general sample, and that average breastfeeding duration was longer for infants born in Baby-Friendly hospitals that had maintained good compliance with the Ten Steps [8]. Analysis of data from 57 hospitals in Oregon, United States, show that breastfeeding rates at two days, and two weeks postpartum increased with the institution's implementation of the Ten Steps [9]. Similarly, results of the United States Infant Feeding Practices II Study indicate that mothers who experienced no Baby-Friendly practices in-hospital were 13 times more likely to stop breastfeeding before six weeks than mothers who experienced six specific Baby-Friendly practices [10]. Widespread implementation of the BFHI has also been associated with increased rates of breastfeeding and exclusive breastfeeding at the regional and national levels [8,12,13].

Although global trends in breastfeeding initiation, duration and exclusivity have generally increased during the years since the introduction of the UNICEF/WHO BFHI, few studies have examined these trends specifically within the context of BFHI activities [13-15]. The purpose of this study is to investigate the contribution of the BFHI to trends in EBF in a group of selected developing countries, through analysis of trends before and after their implementation of the BFHI. We sought to test the hypothesis that overall trends in EBF in countries that implemented the BFHI had increased significantly during the time period after BFHI implementation, compared to the time period prior to the program's launch, and also to estimate the program's contribution to upward trends in EBF in the countries under study.

## Methods

### Data selection

Data were taken from the Demographic and Health Surveys (DHS) from 1986-2006. These nationally representative sample surveys captured population, health and nutrition-related indicators in 72 developing countries (DHS), including prevalence of EBF according to child's age [16]. Use of the DHS limited our analysis to developing countries, but was thought to provide the single best source of consistent data on EBF rates.

First, we selected countries with a minimum of two DHS surveys within the given time frame (the minimum number of data points necessary to establish a trend). A total of 45 countries met this criterion, of which all but three were found to have implemented the BFHI. Due to the lack of "non-BFHI" countries in the data set, we elected to compare trends in EBF before and after implementation of the BFHI, rather than comparing trends between "BFHI" and "non-BFHI" countries.

**Figure 1:**
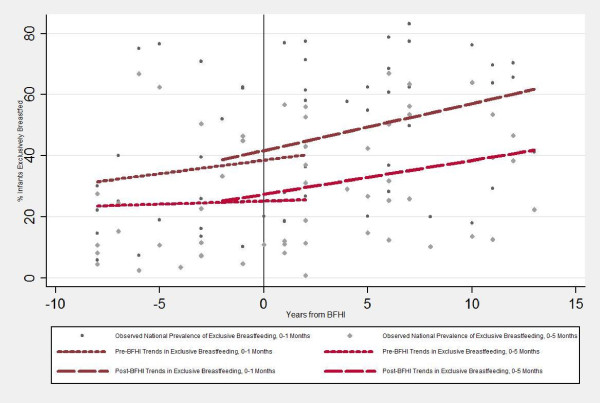
**Trends in exclusive breastfeeding before and after implementation of the Baby-Friendly Hospital Initiative (BFHI)**.

To allow countries to serve as their own comparisons, we selected those with a minimum of two surveys prior to their country's year of initial BFHI implementation, and two after. Few countries had data on EBF available from the year of initial implementation (the "zero" year). In order to capture trends immediately before and after program launch in countries with no "zero" year data, any data point available within two years of BFHI implementation (i.e., within the range -2 to 2) was included in both the before and after data sets for trend. Therefore some countries with only three data points were included in the sample (a decision reflected in the overlapping trend lines seen in Figure 1).

### Outcome variables

The primary outcome variables were the percent of living children under the age of two months and under the age of six months who were exclusively breastfed at the time of survey. EBF as defined by DHS refers to the practice of giving no food or drink other than human milk, measured by 24-hour recall [17]. Although this definition has remained constant, DHS surveys have changed the number of possible responses over time, adding additional categories of "food and drink". These changes occurred in DHS modules used across countries over the years. Adding these additional prompts likely reduces the number of infants who would be considered "exclusively breastfed" over time in all countries. Since this change occurred across all countries, comparison over time remains valid, albeit reducing potential observed increases in EBF rates [18].

### Independent variable

An independent variable was created, "years from BFHI," by subtracting the year of BFHI implementation in the country from the year in which the EBF rate was collected. Negative and positive values denoted data points collected before and after the start of BFHI activities, respectively. The year of BFHI implementation was defined individually for each country as the year in which the first in-country hospital achieved Baby-Friendly status from its national certifying body, as determined from queries to UNICEF country offices and review of UNICEF BFHI reporting data from 1994-2006 [3]. This variable allowed all data points to be considered in relation to their distance from the time of initial implementation, despite countries having implemented the program in different years.

### Statistical analysis

To examine EBF trends, we fitted fixed-effects models in STATA 9.1 (StataCorp, College Station, TX) for the time periods prior to and following program launch. Fixed effects models were chosen to account for the grouping of data points by country and to control for observed and unobserved between-country differences that were fixed over the time period (such as the starting prevalence of EBF immediately prior to program implementation). Linear models were selected based on the positive trends observed in global rates and the relatively small number of data points available.

To compare pre-BFHI and post-BFHI trends in the countries under study, we assessed whether trends were statistically significant during either time period, i.e., whether the slopes of the observed trend pre-BFHI, or the observed trend post-BFHI, was significantly different from zero, and whether the slopes of these observed trends were statistically significantly different from one another.

## Results

### Characteristics of countries in the sample

A total of 14 countries were included in the final sample (Table 1). The latest year of initial BFHI implementation in the sample was found to be 1997. The percentage of in-country maternity hospitals ever certified as Baby-Friendly by 2006 ranged from 3% to 69%, with a median of 17% [3]. The percentage of institutional births (using a 2000-2006 composite measure) ranged from 17% to 97%, with a median of 61% [19].

**Table 1:** Characteristics of countries included in the analysis

**Country (n = 14)**	**Year of BFHI launch**	**Percentage institutional deliveries, 2000-2006 **[19]	Percentage of in-country maternity hospitals ever-certified as Baby-Friendly*
**Bolivia**	1992	57	20
**Brazil**	1992	97	10
**Colombia**	1993	92	37
**Dominican Republic**	1994	95	3
**Egypt**	1993	65	3
**Ghana**	1996	49	13
**Indonesia**	1992	40	5
**Jordan**	1997	97	4
**Kenya**	1992	40	69
**Mali**	1995	38	33
**Niger**	1996	17	49
**Peru**	1994	70	66
**Uganda**	1994	41	3
**Zimbabwe**	1993	68	23

* Percentage of hospitals ever-certified as Baby-Friendly calculated by dividing the total number of hospitals ever certified as Baby-Friendly as determined by 2006 UNICEF reporting records by the total number of in-country maternity hospitals as determined by 1999 UNICEF reporting records [3,4].

### Trends in the rate of EBF pre-BFHI implementation and post-BFHI implementation

When rates of EBF for children less than two months were examined, statistically significant upward trends were observed post-BFHI implementation. Prior to the initiation of BFHI, there was a slow upward trend in EBF, but the slope did not achieve statistical significance (Table 2; Figure 1). Implementation of the BFHI was followed by an average annual increase of 1.54 percentage points in the rate of EBF of infants under two months of age (p < 0.001). Pre-BFHI, the rate of increase was only 0.88 percentage points annually, a difference of 0.66 percentage points. The difference between the slopes of the pre-BFHI and post-BFHI trend lines was not itself statistically significant (95% CI for difference: -0.82, 2.14; p = 0.384).

**Table 2:** Trends in exclusive breastfeeding before and after implementation of the Baby-Friendly Hospital Initiative

**Estimated annual change in the percent of children under two months of age exclusively breastfed (in percentage points)**		
	**Parameter estimate**	**p-value for trend**

**Pre-BFHI**	0.88	0.14
**Post-BFHI**	1.54	< 0.01*

**Estimated annual change in the percent of children under six months of age exclusively breastfed (in percentage points)**		

**Pre-BFHI**	0.20	0.67
**Post-BFHI**	1.11	< 0.01*

*Statistically significant at the p < 0.01 level

Results were similar using EBF for children less than six months as the outcome. Statistically significant upward trends were observed only during the period post-BFHI implementation (Table 2; Figure 1). BFHI implementation was associated with a 1.11- percentage point annual increase in the rate of exclusive breastfeeding in the first six months (p < 0.001), 0.91 points greater than the rate of increase estimated for the period prior to BFHI launch. Again, the difference between the slopes of the pre-BFHI and post-BFHI trend lines was not itself statistically significant (95% CI for difference: -0.22, 2.09; p = 0.131).

## Discussion

### Program impact implications

There is little debate as to the importance of exclusive breastfeeding [1]; however, the effectiveness of programs such as the BFHI has been questioned and there has been a reduction in international support for this program (Labbok M, personal communication from global query carried out in 2006, [3]). Despite the many studies demonstrating the impact of BFHI on breastfeeding rates at the hospital level [6-11], and those that show its impact at the individual country or sub-regional level [8,12,13], no previous study has utilized a multi-country construct based on the actual year of national BFHI implementation, with exclusive breastfeeding as the outcome variable.

Results suggest that among the countries under study, there were no significant upward trends in EBF rates in the years prior to BFHI implementation, but that BFHI implementation was associated with a statistically significant annual increase in rates of EBF in the first two, as well as during the first six, months. The two month rate of increase was higher than the six month rate, as might be expected with an immediate postpartum intervention.

According to the models, a country that implemented the BFHI would experience, on average, a 7.7- and 5.5-percentage point increase in the first two, and first six, months of EBF respectively, over a subsequent period of five years. If improvements in EBF practices are sustained over time, such an increase could provide a significant improvement in child health outcomes. One can estimate the impact of such an increase as follows: based on the accepted estimate that a 51% increase in EBF is needed to reduce child mortality by 13% (i.e., from the 2006 estimate of a 39% prevalence to the 90% prevalence used for calculation of the 13% reduction in child mortality [1]), we estimate that a 5.5% increase in EBF in the first six months has directly reduced annual child mortality by about 1.4%, or prevented about 140,000 deaths. The fact that the slopes of these trend lines did not differ significantly from one another is a call for caution in interpreting these findings. However, the fact that the definitions of EBF became more conservative over time may have blunted the slopes in the later data, reducing the likelihood of achieving significance even if a true increase in positive breastfeeding patterns had occurred.

One strength of our analysis is the use of the "zero" year to re-center all data to the time of country-specific BFHI implementation. This allowed for countries to serve as their own comparisons over time, and for cross-national trends to be considered in relation to the start of BFHI programming, adding strength to the argument that observed trends are derived from BFHI activities. This adds to our understanding of the impact of the BFHI as it was implemented.

### Limitations

The limitations of this study include the reality that the 14 countries analyzed represent a small portion of all developing nations that have implemented the BFHI, and exhibit relatively low rates of hospital certification. In addition, we had no measure of the level of ongoing adherence to the Ten Steps or of the general quality of BFHI implementation over time in this sample. Our results, therefore, are not necessarily reflective of the program's potential to improve breastfeeding rates if implemented on a national or global level.

A serious limitation of any effort to evaluate the effectiveness of the BFHI on cross-national breastfeeding trends is the lack of data collected specifically for this purpose. In this study, we have had to rely on a relatively small number of data points. As such, our study was not powered to detect small differences in trends between pre- and post-BFHI time periods. The limited number of data points also hindered examination of non-linear models that may have provided more insight into the behavior of these trends over time. Our use of overlapping trend lines to compensate for a lack of "zero" year data points, and incomplete information on Baby-Friendly changes that may have been instituted prior to actual certification, further impaired our ability to detect differences between pre- and post-BFHI time periods. We cannot fully predict how access to additional EBF measurements, data from additional countries or information about possible preexisting Baby-Friendly practices may have changed our results.

The use of fixed effects models allowed us to control for the presence of measured and unmeasured confounders that were fixed over the time period studied, but did not control for factors that were variable over the time period, such as demographic changes, shifts in maternal employment patterns, or other breastfeeding promotion programs implemented concurrently with the BFHI. We lacked sufficient information to control for these variables appropriately. With the exception of concurrent public health programming, we would expect most changes over this broad time period, including increased urbanization and women's employment, to have negatively impacted EBF [20]. For this reason, we feel that the observed trends may represent a conservative estimate of the program's potential.

### Future research

If and when sample size and available data permit, additional analyses are needed to consider trends taking into account the percentages of maternity facilities ever-certified as Baby-Friendly, the percent of all births that occur in these facilities, and continued compliance with and investment in the program. Such analyses would help to determine whether a dose-response relationship exists between the level of BFHI programming and trends in EBF over time. Further research is also needed to investigate the existence and impact of other local and national breastfeeding promotion and support programs implemented concurrently with the BFHI.

## Conclusion

Implementation of the international BFHI was associated with a statistically significant annual increase in rates of EBF among infants 0 to 2 months of age and among infants 0 to 6 months of age in the 14 countries studied. Further research is needed to explore fully the impact of the BFHI on cross-national breastfeeding trends, including studies that could better control for individual country's rates of BFHI certification, for whether the practices were maintained, and for the proportion of all births that occurred in Baby-Friendly facilities.

In sum, although the trends following BFHI introduction were not statistically significantly increased from the pre-BFHI trends, and although we were unable to control for all possible confounders, our findings indicate that implementation of the BFHI is associated with positive changes in EBF at a level that would result in improved child health and survival outcomes.

## Competing interests

SA declares no competing interests. ML has served as a consultant to UNICEF and WHO, the implementing agencies of the international Baby-Friendly Hospital Initiative.

## Authors' contributions

SA was responsible for the preparation and analysis of data, wrote the paper with inputs from ML, and contributed to study design. ML was responsible for study concept, and contributed to study design and writing and editing of the final paper.
